# Improving Visualization Methods of Utility-Weighted Disability Outcomes for Stroke Trials

**DOI:** 10.3389/fneur.2022.875350

**Published:** 2022-05-13

**Authors:** Ivie Tokunboh, Eleanor Mina Sung, Fiona Chatfield, Nathan Gaines, May Nour, Sidney Starkman, Jeffrey L. Saver

**Affiliations:** ^1^Department of Neurology and Comprehensive Stroke Center, David Geffen School of Medicine, University of California, Los Angeles, Los Angeles, CA, United States; ^2^Viterbi School of Engineering, University of Southern California, Los Angeles, CA, United States; ^3^Department of Neurology, Division of Interventional Neuroradiology, and Comprehensive Stroke Center, David Geffen School of Medicine, University of California, Los Angeles, Los Angeles, CA, United States; ^4^Departments of Emergency Medicine and Neurology and Comprehensive Stroke Center, David Geffen School of Medicine, University of California, Los Angeles, Los Angeles, CA, United States

**Keywords:** acute stroke, clinical trials, visual display, disability, utility

## Abstract

**Background:**

The modified Rankin Scale (mRS) is the most common endpoint in acute stroke trials, but its power is limited when analyzed dichotomously and its indication of effect size is challenging to interpret when analyzed ordinally. To address these issues, the utility-weighted-mRS (UW-mRS) has been developed as a patient-centered, linear scale. However, appropriate data visualizations of UW-mRS results are needed, as current stacked bar chart displays do not convey crucial utility-weighting information.

**Design/Methods:**

Two UW-mRS display formats were devised: (1) Utility Staircase charts, and (2) choropleth-stacked-bar-charts (CSBCs). In Utility Staircase displays, mRS segment height reflects the utility value of each mRS level. In CSBCs, mRS segment color intensity reflects the utility of each mRS level. Utility Staircase and CSBC figures were generated for 15 randomized comparisons of acute ischemic/hemorrhagic stroke therapies, including fibrinolysis, endovascular reperfusion, blood pressure moderation, and hemicraniectomy. Display accuracy in showing utility outcomes was assessed with the Tufte-lie-factor and ease-of-use assessed by formal ratings completed by a panel of 4 neurologists and emergency physicians and one nurse-coordinator.

**Results:**

The Utility Staircase and CSBC displays rapidly conveyed patient-centered valuation of trial outcome distributions not available in conventional ordinal stacked bar charts. Tufte-lie-factor (LF) scores indicated “substantial distortion” of utility-valued outcomes for 93% (14/15) of conventional stacked bar charts, vs. “no distortion” for all Utility Staircase and CSBC displays. Clinician ratings on the Figural Display Questionnaire indicated that utility information encoded in row height (Utility Staircase display) was more readily assimilated than that conveyed in segment hue intensity (CSBC), both superior to conventional stacked bar charts.

**Conclusions:**

Utility Staircase displays are an efficient graphical format for conveying utility weighted–modified Rankin Scale primary endpoint results of acute stroke trials, and choropleth-stacked-bar-charts a good alternative. Both are more accurate in depicting quantitative, health-related quality of life results and preferred by clinician users for utility results visualization, compared with conventional stacked bar charts.

## Introduction

Visual display of quantitative information can greatly aid understanding of research findings and appropriate action by both experts and laypeople. A general principle of quantitative data display is that particular types of data are most accurately and accessibly depicted using particular types of figural formats (1–3). Accordingly, when a new type of numeric data analysis is developed, it is desirable to also identify or develop preferred formats for figurally displaying the data results.

Recently, a new numeric approach to analyzing primary outcomes in acute stroke trials was advanced-the utility-weighted modified Rankin Scale (UW-mRS) (4). The UW-mRS is a refinement of the modified Rankin Scale (mRS), the most common primary endpoint in acute stroke trials. The modified Rankin Scale is a seven-level scale of global impairment, disability, and handicap; however, its power is limited when analyzed dichotomously and its indication of effect size is challenging to interpret when analyzed ordinally. To address these issues, the UW-mRS has been developed as a patient-centered, linear scale that is both powerful and readily interpretable. Utility weights convert the spacing between the seven ranks on the mRS from arbitrarily uniform intervals to distances that directly reflect patient and societal valuation of each outcome state.

The UW-mRS provides evidence-based weights for different disability health states post-stroke for use in acute treatment trials, as has been recommended for assessing multi-state outcomes in clinical trials for all medical conditions (5–8). In assigning weights to the seven levels of the modified Rankin Scale, UW-mRS provides a simple and efficient technique to rate disability outcomes with greater accuracy than older dichotomous or unweighted polychotomous approaches to mRS analysis. The resulting assessment of disability-related quality of life is not as granular as that afforded by more complex instruments (8, 9), but is often more easily implementable and feasible in large trials (10). The UW-mRS has been supported by leading experts, supported by population-based epidemiologic studies, and endorsed by the Stroke Therapy Academic Industry Roundtable (STAIR) consensus group (5, 11–14). Moreover, it has already been incorporated into acute stroke clinical trial designs, including serving as the primary endpoint in industry, FDA-regulated, and National Institute of Health multicenter pivotal trials (15, 16).

However, appropriate visual displays of UW-mRS results have not yet been explored. Display techniques developed for the ordinal mRS, including generic horizontal stacked bar charts, do not convey utility-weighting information. New or modified figure types are needed to communicate trial UW-mRS results visually in a way that is rapidly interpretable.

We therefore developed and tested two new visual data visualization approaches appropriate for patient-centered weighting of outcomes on ordinal scales across all diseases, including for UW-mRS findings in acute stroke: (1) the choropleth stacked bar chart (a modification of existing stack bar chart data visualizations), and (2) the Utility Staircase.

## Methods

### Visual Display Design

Based on patient population-based and international health-care provider studies, the UW-mRS assigns the each mRS level the following utility values: mRS 0–1.00; mRS 1–0.91; mRS 2–0.76; mRS 3–0.65; mRS 4–0.33; mRS 5–0.00; mRS 6–0.00. (4) The new visual displays appropriate to the UW-mRS were required to convey, in a single image, the following information: (1) frequency of each ordinal outcome in the active treatment group; (2) frequency of each ordinal outcome in the control group; (3) frequency of all possible dichotomized ordinal outcomes in the active treatment group; (4) frequency of all possible dichotomized ordinal outcomes in the control treatment group; (5) value (utility) of each ordinal outcome in the active treatment group; (6) value (utility) of each ordinal outcome in the control group; (7) total value (utility) of all outcomes in the active treatment group; (8) total value (utility) of all outcomes in the control group.

Two types of display meeting these criteria were developed: (1) the choropleth stacked bar chart (CSBC), and (2) the Utility Staircase.

### Construction of Choropleth Stacked Bar Charts

Choropleth stacked bar charts are stacked bar charts that have the added condition that the degree of color intensity assigned to each bar segment is directly proportional to the quantified utility value of the segment's outcome level. As in standard stacked bar charts, each mRS segment length reflects the frequency of patients with the associated mRS value. However, unlike standard bar charts, each mRS segment's color intensity also communicates quantitative information–the utility value of that mRS score. Choropleth stacked bar charts can be made using single colors, with variations in intensity of, for example, greenness, redness, blueness, etc., or two color gradients, with quantified hue transitions from one color at one pole to a second color at the other pole. For this study, we created green CSBCs. Using RGB color model settings (which maps to the red, green, and blue optimum wavelengths of the three types of human retinal cone cells), red and blue inputs were set to zero, and the green input was varied proportionately to the utility value of each mRS segment. Accordingly, mRS 0 was assigned a green value of 255 (=255 × 1.0), mRS 1 a green value of 232 (=255 × 0.91), mRS 2 a green value of 194 (=255 × 0.76), mRS 3 a green value of 166 (=255 × 0.65), mRS 4 a green value of 84 (=255 × 0.33), and both mRS 5 and 6 a green value of 0 (=255 × 0.00, resulting in black coloration for segments five and six). Use of color to convey mRS level severity is already often employed in stacked bar charts of the mRS, but by employing arbitrary degrees of variation in hue rather than exactly measured RGB/brightness values corresponding to the utility weight.

### Construction of Utility Staircase

Utility Staircase charts are derived from the starting point of standard stacked bar charts by adding the condition that the height of assigned to each bar segment is directly proportional to the quantified utility value of the segment's outcome level. As in standard stacked bar charts, each mRS segment's horizontal width reflects the frequency of patients with the associated mRS value. However, unlike standard bar charts, each mRS segment's height also communicates quantitative information–the utility value of that mRS score.

Along the x-axis of the Utility Staircase figure, the chart shows the proportion of patients with outcomes occurring in each mRS level. Along the y-axis, the chart shows the utility of each mRS segment, from zero to one. The total utility across the study population yielded by a treatment arm is visually conveyed by the total area of that treatment group's color field on the graph. The Utility Staircase plots have greater heights toward the left of the figures, reflecting the higher utility values of lower mRS scores, and lower heights toward the right of the figures, reflecting the lower utility of higher mRS scores. When more good outcomes occur, the higher utility value segments expand in width rightward, to take up more and more of the available figural space (the “utility space”). As a result, the area of rightward and upward shift in color field associated with better outcomes in a treatment group exactly quantifies the total utility value gained at the treatment group level. At the extremes, a treatment that resulted in fatal outcomes for all patients would yield an empty figural space, reflecting zero utility at the group level, while a treatment that resulted in fully normal mRS 0 values for all patients would yield a color rectangle occupying the entire available figural space, reflecting perfect utility at the group level. The utility value for the control group is conveyed by a gray color field. When there is unidirectional benefit, the utility value for the experimental group is conveyed by the additive combination of the gray and green color fields. When there is unidirectional harm, the utility value for the experimental group is conveyed by the subtraction of the red color field from the gray color field. When there are bidirectional effects, the utility value for the experimental group is conveyed by the additions of the green color field and subtractions of the red color field.

In the Utility Staircase generated for this study, the control group outcome distribution is assigned a gray color. The active treatment group is assigned a green color in regions where outcomes are more favorable than control and a red color in regions where outcomes are less favorable than control. If there were no difference whatsoever between the treatment groups in frequency of mRS outcomes at every level, the two treatments would overlap exactly and only the gray color would be visible. The total area of the green regions minus the red regions in a figure visually and quantitatively conveys the total, population-level, increase in utility conferred by the active treatment.

### Analysis of Figural Accuracy

Formal analysis was performed of the degree to which the standard stacked bar charts, the choropleth stacked bar charts, and the Utility Staircase charts quantitatively captured the health-utility value of clinical trial outcomes. When display elements were disproportionate to the numeric value of utility, the degree of distortion was quantified using the Utility Distortion Factor (UDF), a variation of the Tufte lie factor (LF):(1) UDF = (size of cumulative group utility treatment effect shown in graphic) ÷ (size of cumulative group utility treatment effect shown in data). As recommended by Tufte, values >1.05 or <0.95 were considered to indicate substantial distortion. Additional details of the figural accuracy analysis methods are provided in Supplementary Methods Text 1.

### Testing User Preferences

We constructed a formal rating scale to assess clinician preference among the three figural display options: the Showing Utility trial Result Findings - Clinician Assessment Scale (SURF-CAS). SURF-CAS scale items were adapted from the Practitioner Opinion (Acceptance) Survey and the Preparation for Decision Making Scale (17, 18). The resulting SURF-CAS instrument has five Likert scale items, asking clinicians to indicate which of two figural displays of trial utility results is: (1) overall easier to understand; (2) better visualizes the value a treatment group outcomes: (3) better conveys the difference in value of treatment outcomes; (4) more closely accords with how the clinician thinks about the value of study outcomes, and (5) better help the clinician recommend a treatment based on what matters to patients. (Supplementary Figure 1) for each scale item, the score ranges from +2 (Strongly Agree), +1 (Agree), 0 (Neutral), −1 (Disagree), and −2 (Strongly Disagree). The total SURF-CAS Scale score accordingly ranges from +10 (strong preference for first figure in pair) through 0 (neutrality between figures) to −10 (strong preference for second figure in pair). The SURF-CAS was administered to a purposive sample of five clinicians selected to represent different types and levels of training, including a senior faculty Emergency Medicine and Neurology physician, a mid-career faculty noninvasive Vascular Neurologist, a junior faculty Interventional Neurologist, a Vascular Neurology fellow, and a stroke center Nurse-Coordinator. After a brief orientation to the task and figure types, all raters assessed how well utility outcome information was conveyed by standard stacked bar charts, CSBCs, and Utility Staircase displays showing the results of the same four randomized trials, encompassing a positive endovascular thrombectomy trial (DAWN), a neutral endovascular thrombectomy trial (IMS 3), a positive intravenous thrombolysis study (NINDS-tPA Study), and a positive hemicraniectomy trial (DESTINY 2).

## Results

**Figure 1:**
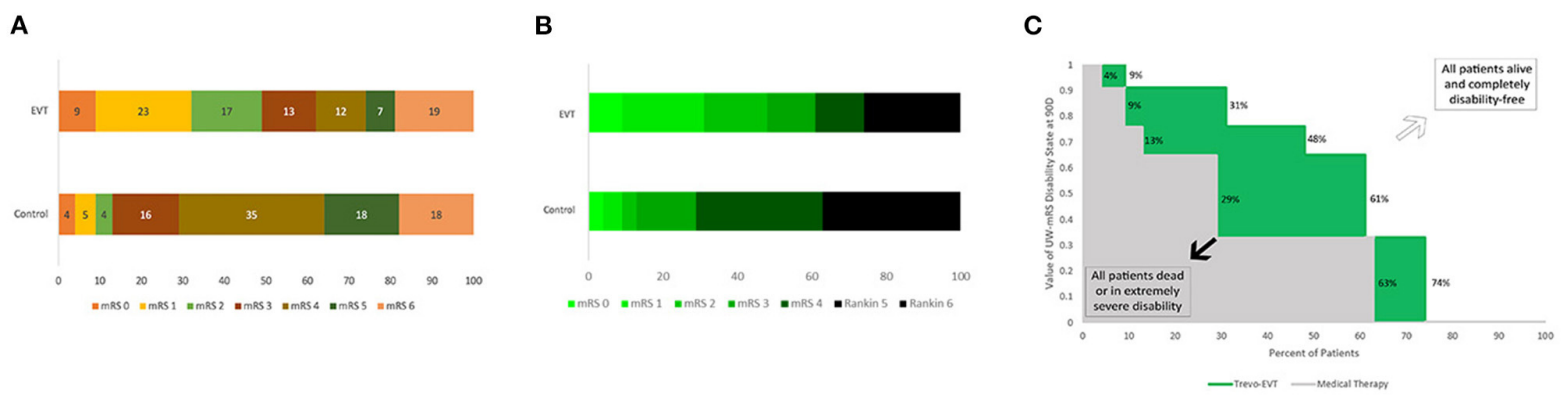
Comparative Standard Stacked Bar Chart, Choropleth Stacked Bar Chart, and Utility Staircase Chart Displays. Outcomes at 3 months on the ordinal modified Rankin Scale from the same trial, DAWN, are shown using each figural approach: **(A)** Standard stacked bar chart–display visually conveys mRS rank frequencies (bar segment widths), but does not explicitly visually communicate utility values; **(B)** Choropleth stacked bar chart–display visually conveys both mRS rank frequencies (bar segment widths) and mRS rank utility values (degree of green intensity); and **(C)** Utility Staircase chart–display visually communicates both mRS rank frequencies (row widths) and mRS rank utility values (column heights). In addition, display directly communicates utility value of the two treatment groups: utility value of control treatment–area size of gray coloration; utility value of active treatment–area size of combined gray and green coloration; utility gain with active treatment–area size of green coloration.

The three visual display types, stacked bar charts, choropleth stacked bar charts, and Utility Staircase charts, are shown side-by-side in Figure 1, with each chart type depicting results from the same trial, DAWN, a study in which the UW-mRS was the lead primary endpoint. (15).

### Figural Accuracy in Displaying Health-Utility Findings

For the standard stacked bar charts, among the 15 RCTs analyzed, the UDF values ranged from 0.53 to 1.38. The absolute error ranged from 0.02–0.47, with mean absolute error 0.21 (±0.15). Substantial distortion in the depiction of health-utility outcomes was present in 14/15 (93%) displays. In contrast, for both CSBC and Utility Staircase Displays, UDF values were uniformly 1.0 for all charts, without any occurrence of substantial distortion in the depiction of health-utility outcomes.

### Clinician Ratings of Figural Effectiveness

The results of expert physician and nurse clinician ratings comparing the three display types are shown in Table 1. Responses had no missing data. Ratings showed trends to indicating that utility information encoded in row height (Utility Staircase Display) and conveyed in segment hue intensity (CSBC) were superior to conventional stacked bar charts.

**Table 1:** Clinician preferences among visual displays of trial utility results.

SURF-CAS Item^*^	**CSBC vs. SBC**	**Utility staircase vs. SBC**	**Utility staircase vs. CSBC**
1. Understanding	0.30 (±0.45)	0.30 (±1.30)	0.20 (±1.36)
2. See utility of group outcomes	0.75 (±0.83)	1.45 (±0.74)	0.35 (±1.45)
3. See differences between groups in utility	0.95 (±0.84)	1.60 (±0.65)	0.60 (±1.52)
4. Compatible with way I think	0.75 (±0.61)	1.15 (±1.14)	0.60 (±1.34)
5. Helps me recommend	0.70 (±0.45)	1.00 (±1.22)	0.60 (±1.34)
Total score^**^	3.45 (±2.56)	5.50 (±4.67)	2.35 (±6.82)

*SURF-CAS-Showing Utility Results Figures- Clinician Assessment Scale; CSBC-Choropleth stacked bar chart; SBC-stacked bar chart. Values are mean (± standard deviation)*.

* *Scores for each item range from +2 (strong preference for first figure in pair) to –2 (strong preference for second figure in pair), with zero indicating neutrality. Ratings were obtained from a purposive sample of five clinicians selected to represent different types and levels of training, including a senior faculty Emergency Medicine and Neurology physician, a mid-career faculty noninvasive Vascular Neurologist, a junior faculty Interventional Neurologist, a Vascular Neurology fellow, and a stroke center Nurse-Coordinator*.

** *Total scores range from +10 (strong preference for first figure in pair) to−10 (strong preference for second figure in pair), with 0 indicating neutrality. P values: CSBC vs. SBC p=0.10; Utility Staircase vs. SBC, p = 0.13; Utility Staircase vs. CSBC, p = 0.63*.

### Utility Staircase Trial Displays for Diverse Trials

**Figure 2:**
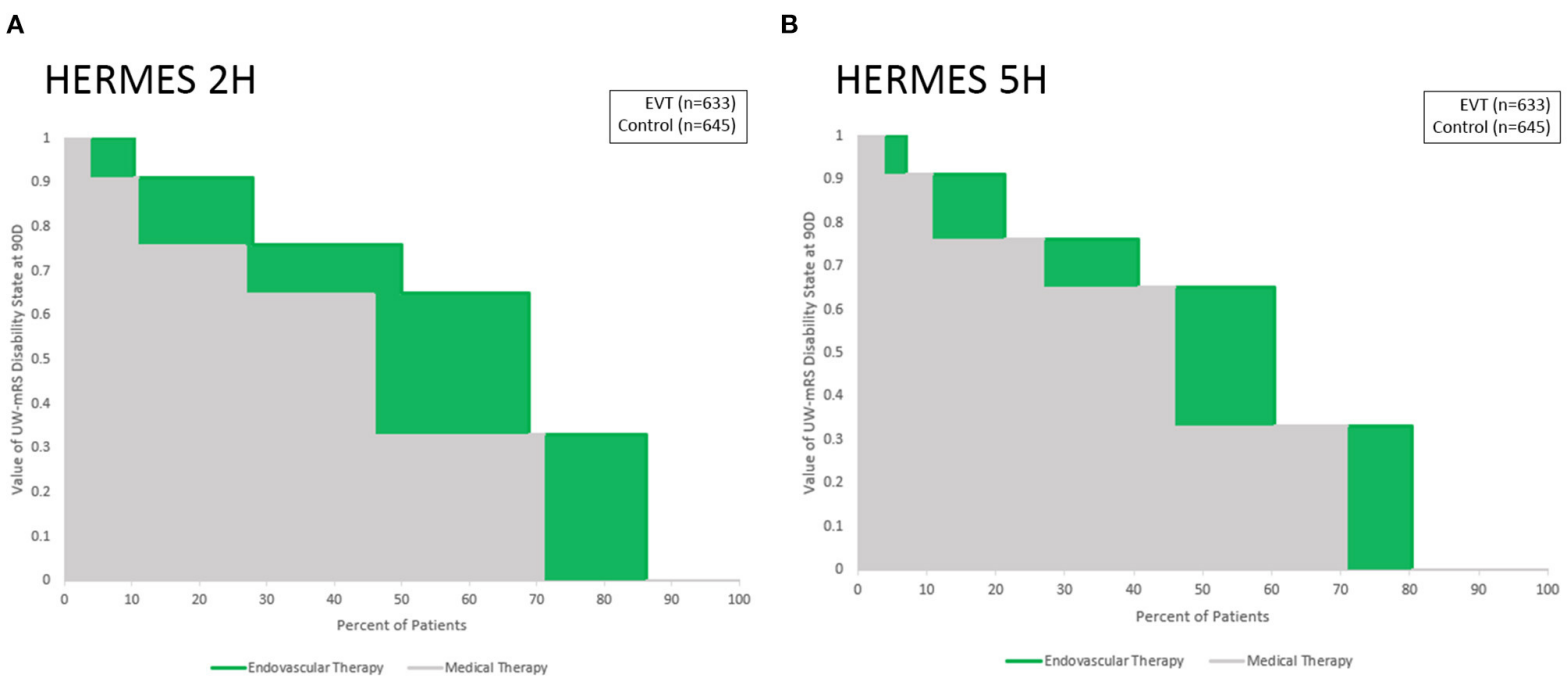
Utility Staircase charts demonstrating effects of different onset to treatment times for endovascular thrombectomy. Outcomes at 3 months on the UW-mRS are shown for: **(A)** last known well to puncture of 2 h, and **(B)** last known well to puncture of 5 h. Medical therapy outcomes (gray) are similar in both time periods, while endovascular thrombectomy outcomes have greater differential additional value (green) with earlier treatment start.

**Figure 3:**
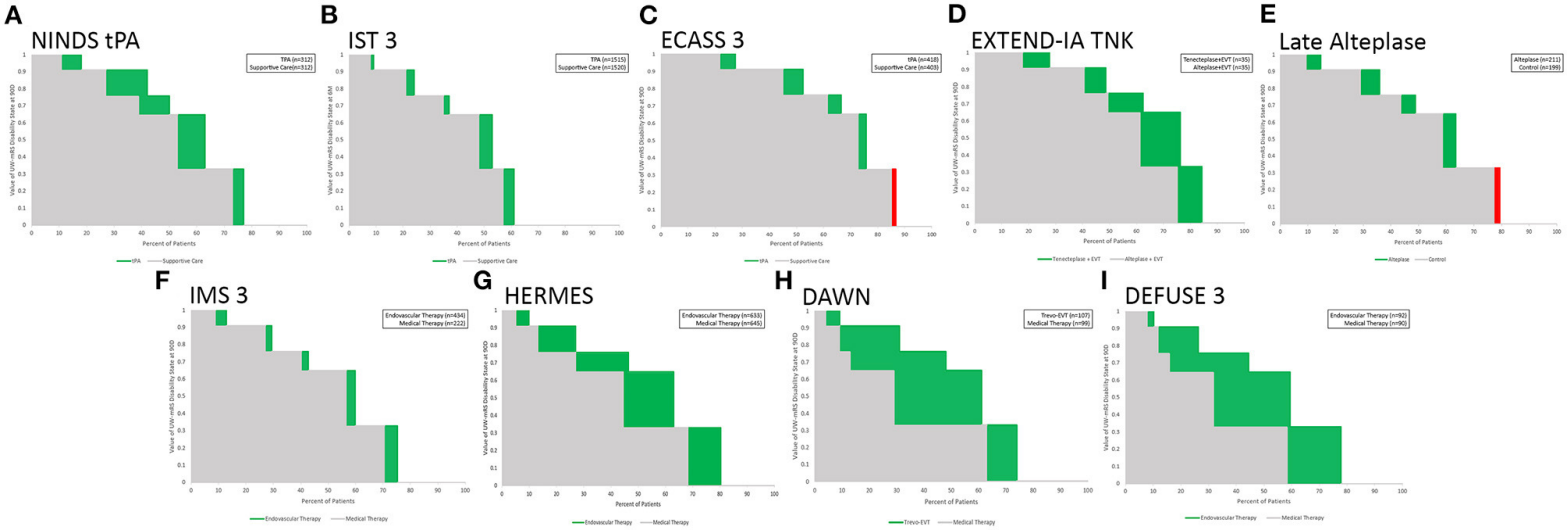
Utility Staircase charts showing nine trials and meta-analyses of reperfusion therapy for acute ischemic stroke. Top row: intravenous fibrinolysis trials, including: **(A)** IV tPA under 3 h; **(B)** IV tPA up to 6 h; **(C)** IV tPA in 3–4.5 h;**(D)** IV tenecteplase vs IV tPA in large vessel occlusion patients up to 4.5 h, and **(E)** IV alteplase in imaging selected patients after 4.5 h. Bottom row: endovascular reperfusion trials, including: **(F)** early generation techniques;**(G)** second generation mechanical thrombectomy largely in early time windows **(H)** second generation mechanical thrombectomy with imaging selection 6–24 h after onset, and **(I)** second generation mechanical thrombectomy with broader imaging selection 6–16 h after onset.

**Figure 4:**
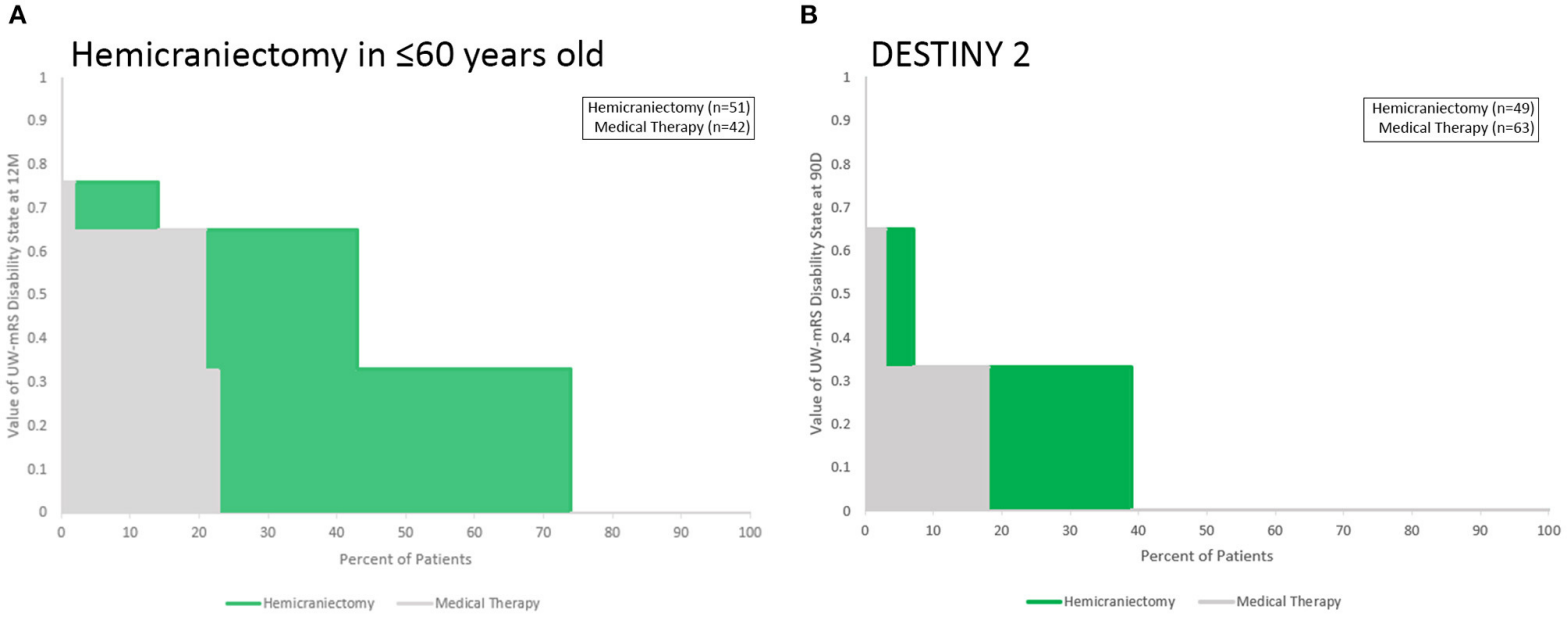
Utility Staircase charts showing trials data for hemicraniectomy for malignant acute ischemic stroke. **(A)** Among patients up to age 60 years old; **(B)** among patients over 60 years old. The greater utility benefit among younger patients is readily apparent, as is the overall poor health-related quality of life outcomes in both control and treated groups in both age ranges (both gray and green color fields hew toward the lower left portion of the rectangular achieved utility space).

The Utility Staircase Displays for the analyzed trials demonstrate the applicability of this figural format to diverse treatment modalities, patient groups, and outcome scales. For acute ischemic stroke with the UW-mRS as the primary outcome, displays readily visually convey key utility findings related to effects of later onset to treatment time for time-urgent therapies (Figure 2); different types of pharmacologic and endovascular reperfusion therapy (Figure 3); and effects of hemicraniectomy in instances of malignant acute ischemic stroke for patients both under and over 60 years old (Figure 4).

Several aspects of these Figures exemplify the insights conveyed by the Utility Staircase Displays. For example, in Figure 3, in the top row, comparison of the first three figures showing different intravenous alteplase trials shows at a glance the greater overall utility benefit provided by earlier treatment (A vs. B and C) and the better control group utility outcome starting point resulting from milder deficits at entry (C vs. A and B). In the bottom row, comparison of figures F through I shows at a glance the great utility benefit from second generation compared with early endovascular reperfusion techniques (G-I vs. F) and the substantially equivalent degree of utility benefit with treatment in imaging selected, late patients vs. time-selected early patients (H, and I vs. F). In addition, comparison of the panels in top vs. bottom rows shows at a glance the overall better control group starting point of health-related quality of life outcome, resulting from milder deficits at entry in the broader population of IV fibrinolysis patients (A–E) compared with the narrower population of large vessel occlusion patients (F–I).

Supplementary Figure 2 demonstrates two additional aspects of the Utility Staircase method. First, it shows applicability to a different disease subtype, acute intracerebral hemorrhage rather than ischemic stroke. Second, it exemplifies how the Utility Staircase approach displays results when an experimental treatment tends to have an adverse, rather than favorable, effect on health-related quality of life. The plot of INTERACT two shows a favorable point estimate for moderate vs. mild blood pressure lowering, with the magnitude of enhanced utility outcomes displayed in green. Conversely, the plot of ATACH 2 shows an unfavorable point estimate for intensive versus moderate blood pressure lowering, with the magnitude of reduced utility outcomes displayed in red.

In Supplementary Figure 3, the plot demonstrates that the Utility Staircase figural methodology is generalizable across all scales to which utility values can be assigned to measure scores, not just the seven level mRS scale. This figure shows health-related quality of life outcomes arising from the four level 3SQ ordinal scale, the primary outcome measure in the IST1, and CAST mega-trials of aspirin in early ischemic stroke. The graphic also readily demonstrates the small absolute magnitude of utility benefit conferred by early aspirin vs. no aspirin, compared with the larger treatment effects in other figures. Supplementary Figure 4 shows further elements that can optionally be added to Utility Staircase Displays: adding the actual n values or percent values for each mRS in each treatment arm as embedded data labels. This approach provides more granular information regarding the numeric strength of the underlying evidence. However, it draws reader attention back to the ordinal aspects of the data and away from the utility-weighted aspects of the data that are indicated by the color field areas in their whole, not in part.

**Figure 5:**
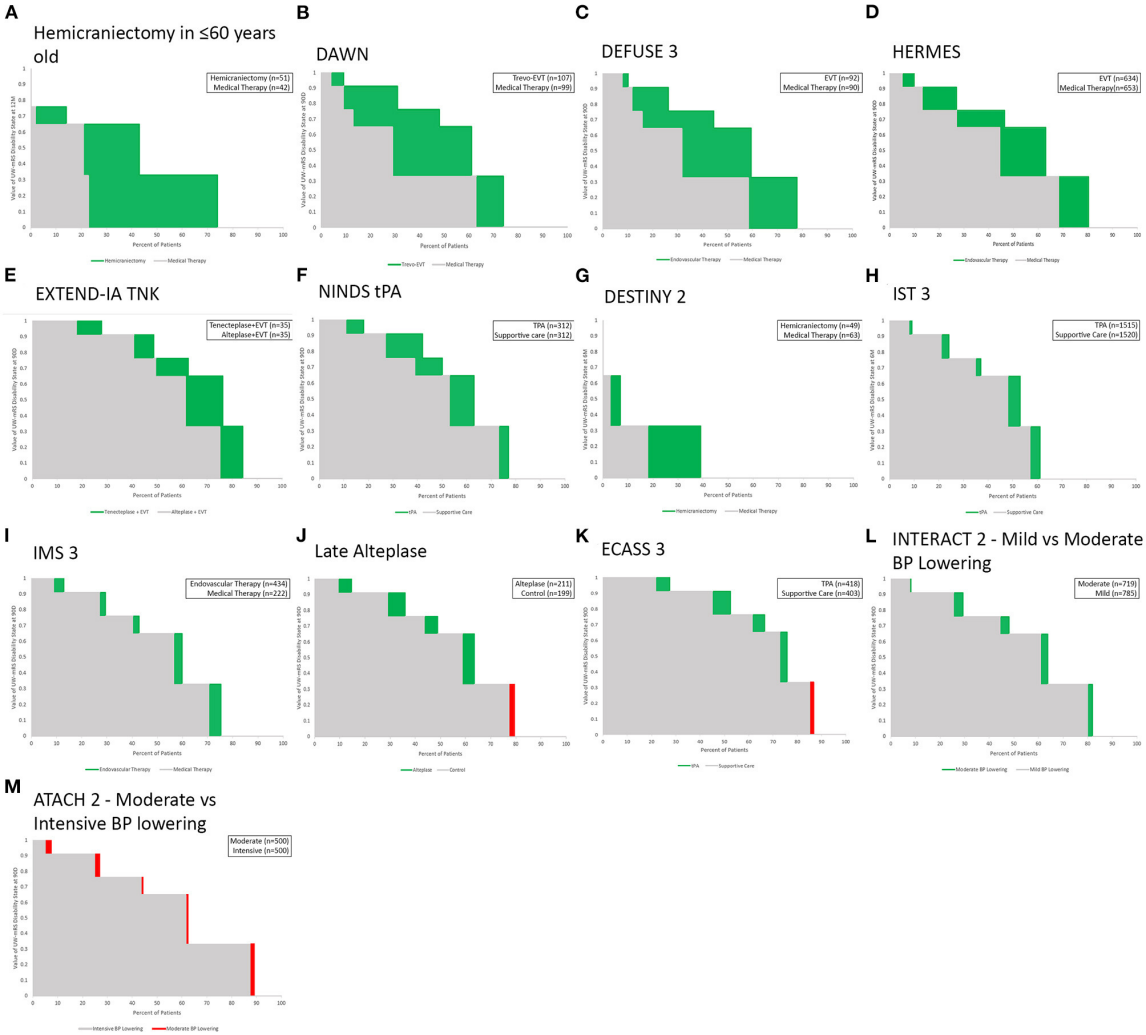
**(A–M)** General comparative display of Utility Staircase plots for 13 RCTs and meta-analyses of treatments for acute ischemic and hemorrhagic stroke that used the mRS as a primary endpoint. Charts are ordered, left to right and then top to bottom, by each study's point estimate of the active intervention's overall magnitude of effect in increasing health-related quality of life.

Figure 5 shows a general comparison of 13 RCTs of diverse therapies for varied subtypes of acute ischemic and hemorrhagic stroke, ordered by the point estimate of magnitude of utility benefit on the UW-mRS of the active intervention. The greatest utility gains are conferred by endovascular thrombectomy with modern devices and by hemicraniectomy for malignant infarction in young patients, the latter accruing benefit in the lower quadrant of utility space vs. thrombectomy accruing benefit in the mid-range of utility space. The least utility gains are conferred by older endovascular thrombectomy techniques and late intravenous fibrinolysis for ischemic stroke, and blood pressure lowering for intracerebral hemorrhage, the latter three associated with fairly good utility outcomes in control groups, tending toward the upper quadrant of the utility space.

## Discussion

The Utility Staircase Chart and Choropleth Stacked Bar Charts are novel approaches to visualizing health-related quality of life outcomes in randomized clinical trials. These displays are appropriate to figurally convey results of trials using as a primary or auxiliary endpoint any ordinal or continuous scale having levels or scores convertible to health-related utility values, including the utility-weighted modified Rankin Scale recently developed and deployed for acute stroke trials. In the current study, both the Utility Staircase and CSBC plots outperformed conventional stacked bar chart displays, objectively in depicting health-related quality of life results without quantitative distortion and subjectively in clinician ratings of clarity and usefulness for decision-making. Moreover, among the two developed display types, clinicians nominally rate the Utility Staircase Display superior to the CSBC in clarity and decision-making value.

The optimal display approaches for different types of medical data has been the subject of extensive investigation over the past two decades, including development of displays appropriate for binary, ordinal, linear, categorical, and multidimensional data, including bar charts, line plots, scatter plots, trellis plots, spie charts, radar charts, and person-icon arrays (1–3, 19–22) As far as we aware, prior studies have not addressed developing and assessing data visualizations that are appropriate for conveying health-related quality of life outcomes and outcome differences over the full outcome spectrum among intervention and control groups in randomized clinical trials. In the last two decades, clinical trial designs have increasingly incorporated patient-reported outcome measures and health-related quality of life endpoints, so that trial findings can be assimilated into practice based not solely upon disease-specific measures of variable clinical import, but also upon effects on life value that are clearly meaningful to patients, families, and society (23, 24). Accordingly, figural display formats appropriate for health utility outcome distribution data, like the Utility Staircase and CSBC, could be of wide applicability.

While quantitatively accurate Choropleth Stacked Bar Charts were not developed prior to the recent DAWN trial (15), a variety of approaches have previously been taken by researchers to assigning color values to modified Rankin Scale outcomes in stacked bar charts. In some study reports, the mRS stacked bar chart is shown without any color or shade variation (25), so that the color/shading channel is not used to convey value information. In other trial publications, the mRS stacked bar chart is shown with color or shading variations that perform various roles. These approaches vary widely. In some reports, each mRS cell is given a graded variations in shading or color intensity that conveys value information qualitatively, but not quantitatively, reinforcing the message, already communicated by cell position in the stacked bar chart, that the outcomes are ordered, but not providing added information. This qualitative reinforcement is most often monochromatic but with variation in shading/intensity of the single color (26, 27). Different groups have chosen directionally opposite patterns of shade variation, some depicting better outcomes as lighter (26, 27), and others depicting better outcomes as darker (28, 29). Other clinical trial reports have used cell coloring to embed a dichotomous analysis of the mRS in the figure, coloring good outcome cells (e.g., mRS 0,1,2) with one color and bad outcome cells with another color (e.g., mRS 3,4,5,6) (30). In addition, color choices by different group have reflected disparate implicit color metaphors. such as using funereal black or gray for the mRS 6 fatal outcome level, or traffic light-based green spectrum colors for better outcomes (e.g., mRS 0, 1, 2), yellow spectrum colors for intermediate outcomes (e.g., mRS 3, 4), and red spectrum colors for poor outcomes (e.g., mRS 5, 6). (31) At the extreme, some clinical trial reports have used alternating colors (32), or apparently random unique colors, (33) for the different mRS ranks, making the mRS levels more visually distinct, but doing so in a manner that somewhat undermines even the simple information about their relative value conveyed by their order in the stacked bar. In all of these previous approaches, color and color intensity selections were made qualitatively, and did not align with the actual health utility value of each of the mRS levels.

While the CSBC displays tested in this study overcame this limitation by having each mRS level's numeric utility value precisely determine its color intensity, the use of color to convey utility information still had limitations. First, readers with color blindness will not be able to appreciate all information conveyed in the color channel. This drawback can be mitigated by preparing alternate figures in which color intensities are varied within the spectrum of multiple colors (34). For this study, we prepared figures with variations in the color green, but the same approach could be used employing variations in blue, yellow, red, or other colors, or simply in gray-black, so that readers with different forms of color blindness would still have access to figures with variations visible to them. But another limitation is that human subjective responses to color intensity variation do not exactly track objective changes in red-green-blue color scale variation (35), and are culturally-influenced so non-universal (36). As a result, even color values exactly matched to mRS utility values may not evoke subjective responses in readers matched to the utility values. These limitations of color signaling may be one reason that physician and nurse clinicians did not nominally value the CSBC figures as highly as the Utility Staircase figures.

In contrast, the Utility Staircase figures' use of cell height to convey value is a psychophysically more sound approach to conveying quantitative information. Degree of linear extension vertically (height) is readily discriminated by humans in a manner that is highly accurate and relatively culturally invariant (37). Moreover, it accords with the manner in which mRS stacked bar charts are already conveying information about the frequency of the different mRS outcomes–degree of linear extension horizontally (width). For each mRS level, cell width (outcome frequency) and cell height (outcome value) combine to yield cell area as direct, readily appreciable, quantitative indicator of the contribution of that outcome to the treatment group's total achieved utility. The combination of frequency along the x axis and value along the y axis yields a figure in which a perfect outcome would be a rectangular color field occupying the entire graphical space (all patients achieving mRS 0). This approach is similar that widely used in figural displays of receiver operating curves evaluating diagnostic test performance. There too the directly measured variable is conveyed along the x axis (biomarker amount) and its clinical value is conveyed along the y axis (degree of combined sensitivity and sensitivity), yielding a display in which a perfect outcome would be rectilinear lines going straight up to the top of the chart and then straight across.

Both the Utility Staircase figure and the Choropleth Stacked Bar Chart are applicable to all ordinal scales with outcome levels that can be mapped to health-related quality of life. This generalizability was shown in the current study by creating Utility Staircase and CSBC figures not only for trials reporting results on the seven-level modified Rankin Scale, but also to a trial reporting results on the four-level 3SQ scale. The same approach can be used for the diverse ordinal scales used in clinical trials of varied disease states with that have discrete, ordered outcome states, including multiple sclerosis (e.g., expanded Kurtzke disability scale), traumatic brain injury (e.g., expanded Glasgow outcome scale), cardiac arrest (e.g., expanded Glasgow outcome scale), cancer (e.g., Karnofsy performance scale), and congestive heart failure (e.g., New York Heart Association Functional Classification). In principle, the Utility Staircase display can be similarly deployed to depict outcome values for disease-specific outcome scales that have continuous, rather than ordinal, outcome distributions, as long as a valid method is available to convert the disease-specific outcome values to health-related quality of life values.

This study has limitations. First, though the clinician raters assessing the different figural displays were purposively sampled to include diverse stakeholders, including nurses, physician-trainees, junior, mid-career, and senior faculty, and different specialties, all were from a single institution and certain specialties salient to acute stroke care, such as neurosurgeons, were not represented. Assessment is desirable of display type preference in numerically more individuals, additional stakeholder groups and geographic regions, potentially by online administration. Similarly, although Utility Staircase and CSBC displays were assessed for 14 different trials evaluating varied types of interventions (medical, endovascular, and open surgical), for different disease stages (acute and early secondary prevention), and different stroke subtypes (ischemic and hemorrhagic), further evaluation would be helpful in trials assessing other treatment modalities (e.g., rehabilitation) and additional stroke subtypes (e.g., subarachnoid hemorrhage). Second, the raters could not be objectively tested to evaluate improvements in understanding before and after figure review, as they were being asked to compare three figures precluding assessment of the independent knowledge contribution of any one figure type. Instead, they provided subjective ratings of the comparative degree to which they found the figures helpful. Future studies of knowledge acquisition with the highest-rated figure type, the Utility Staircase are desirable. Third, the standard stacked bar chart visualization assessed used only right-to-left placement to convey outcome levels visually. While this approach is used in the literature, it is also common to add use of color but employing arbitrary degrees of variation in hue rather than exactly measured RGB/brightness values corresponding to the utility weight. Rater preferences for the chloropleth stacked bar chart and Utility Staircase over the standard stacked bar chart likely would have been less if an arbitrarily color-graded standard bar chart was the comparator. Fourth, the displayed figures employ green color for positive and red color for negative outcomes. This strategy has the advantage of being symbolically-based upon the worldwide foundation of the use of green for go and red for stop at traffic lights (38). However, this approach may cause perceptual difficulty for individuals with red-green color blindness, for whom other color options may be employed.

## Conclusions

Utility Staircase displays are an efficient graphical format for conveying utility weighted–modified Rankin Scale primary endpoint results of acute stroke trials, and choropleth stacked bar charts a good alternative. Both are more accurate in depicting quantitative, health-related quality of life results, and preferred by clinician users for utility results visualization, compared with conventional stacked bar charts.

## Data Availability Statement

The raw data supporting the conclusions of this article will be made available by the authors, without undue reservation.

## Ethics Statement

Ethical review and approval was not required for the study on human participants in accordance with the local legislation and institutional requirements. Written informed consent for participation was not required for this study in accordance with the national legislation and the institutional requirements.

## Author Contributions

IT: data analysis and writing of first draft. ES: data analysis and writing of second draft incorporating additional data. FC, NG, MN, and SS: served as expert raters and provided critical review and comments on manuscript. JS: study conception, data analysis and provided critical review and comments to manuscript. All authors contributed to the article and approved the submitted version.

## Funding

This research and open access publication fees were supported by the UCLA Health System. The funder had no role in the design or interpretation of study results.

## Conflict of Interest

The authors declare that the research was conducted in the absence of any commercial or financial relationships that could be construed as a potential conflict of interest.

## Publisher's Note

All claims expressed in this article are solely those of the authors and do not necessarily represent those of their affiliated organizations, or those of the publisher, the editors and the reviewers. Any product that may be evaluated in this article, or claim that may be made by its manufacturer, is not guaranteed or endorsed by the publisher.
