# Supplementary material for: Improving Visualization Methods of Utility-Weighted Disability Outcomes for Stroke Trials
Source: Front Neurol. 2022 May 13;13:875350. doi: 10.3389/fneur.2022.875350 (PMC9136165; doi:10.3389/fneur.2022.875350)
Supplement: Supplementary file 1 [file Data_Sheet_1.docx]

Supplementary Material

# Supplementary Methods

**Supplementary Methods Text 1: Additional details of the figural accuracy analysis methods**

Analyses were performed of visual displays for 15 completed randomized trials and trial meta-analyses. The 15 trials were purposively selected to include: 1) diverse intervention types, including pharmacologic, endovascular, and open surgical; 2) diverse acute stroke disease states, including both acute ischemic stroke and acute intracerebral hemorrhage; 3) long-term outcomes reported primarily on the mRS ordinal scale (14 trials), but also including 1 trial using a different primary outcome ordinal functional scale, to show generalizability of the displays; and 4) studies of treatments salient to current clinical practice. The roster of selected studies included trials of fibrinolysis, endovascular reperfusion, blood pressure moderation, antiplatelet therapy, and hemicraniectomy.

For the modified Rankin Scale, utility values of the 7 outcome levels were taken from the UW-mRS. For the one trial that employed the “two simple questions” technique to assign disability outcome, the results were converted into a “3 Simple Questions (3SQ) scale. The added third question was whether the patient was alive or dead. The resulting 4 possible outcome states were mapped to the mRS extending a prior approach,^17^ “Complete recovery” – mRS 0; Incomplete recovery but not needing help from another person for everyday activities – mRS 1-2; needing help from another person for everyday activities – mRS 3-5; death – mRS 6. The 3SQ utility values were then assigned by weighted averaging of mRS level values and mRS level frequencies in a multicenter study of consecutively presenting ischemic stroke patients.^18^ The resulting 3SQ utility values were: level 1 – 0.96; level 2 – 0.76; level 3 – 0.41; and level 4 – 0.00.

To calculate the UDF for standard stacked bar charts, we considered that, since these apportion equal psychophysical visual value to each mRS segment, they effectively assign each step in the mRS scale an equal increment of utility value. The display implicitly communicates that: an mRS score of 0 has a utility of 1.0; an mRS 1 has utility value of 0.83; an mRS 2 has utility value of 0.67; an mRS 3 has a utility value of 0.50; an mRS of 4 has a utility value of 0.33; an mRS value of 5 has a utility value of 0.17, and an mRS value of 6 has a utility value of 0.0. Accordingly, the utility value of treatment group outcomes conveyed by standard stacked bar charts is equal to the treatment group’s mean mRS value, normalized and inverted from the 0 to 6 mRS range to the 0 to 1 utility range. UDFs for each trial were were determined by dividing the mean group differences in utility when valuing each mRS level as equally spaced (visual depiction) by the mean group differences in actual UW-mRS utility values.

# Supplementary Figures

| In general, display format B compared with A was: | Strongly Agree | Agree | Neutral | Disagree | Strongly Disagree |
| --- | --- | --- | --- | --- | --- |
| Easier for me to understand |  |  |  |  |  |
| Easier for me to see the utility (worth) of outcomes in each treatment groups |  |  |  |  |  |
| Easier for me to see the difference in utility (worth) of outcomes between the treatment groups |  |  |  |  |  |
| Is more compatible with the way I think about the value of treatment outcomes |  |  |  |  |  |
| Helps me know that I can recommend a treatment based on what matters most to patients and families |  |  |  |  |  |

**Supplementary Figure 1.** The Showing Utility Trial Result Findings – Clinician Assessment Scale (SURF-CAS Scale)


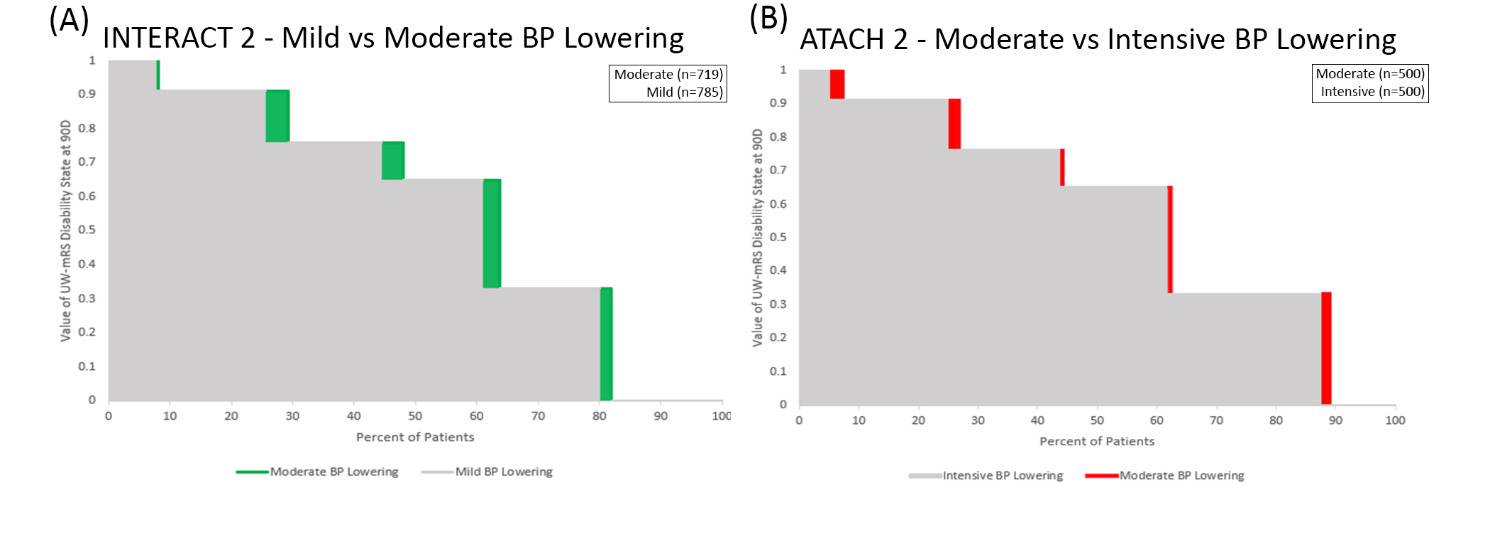


**Supplementary Figure 2.** Utility Staircase displays showing of blood pressure lowering in intracerebral hemorrhage. A) Mild versus moderate blood pressure lowering; B) Moderate versus intensive blood pressure lowering. Green field in A indicates magnitude of improved utility outcome point estimate with moderate versus mild blood pressure lowering. Red field in B indicates magnitude of reduced utility outcome point estimate with intensive versus moderate blood pressure lowering.


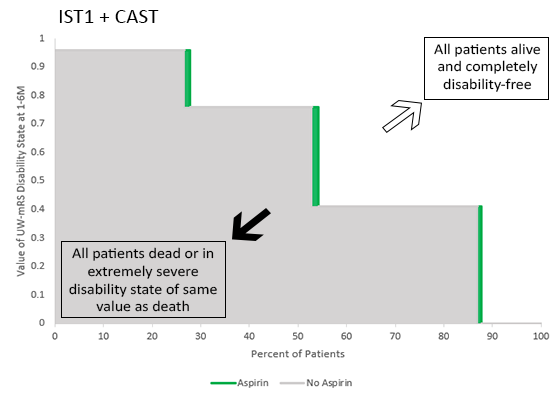


**Supplementary Figure 3.** Utility Staircase plot showing effect of aspirin in early ischemic stroke across the two mega-trials, IST1 and CAST. Displays show health-related quality of life results for the 3SQ ordinal outcome scale.

**
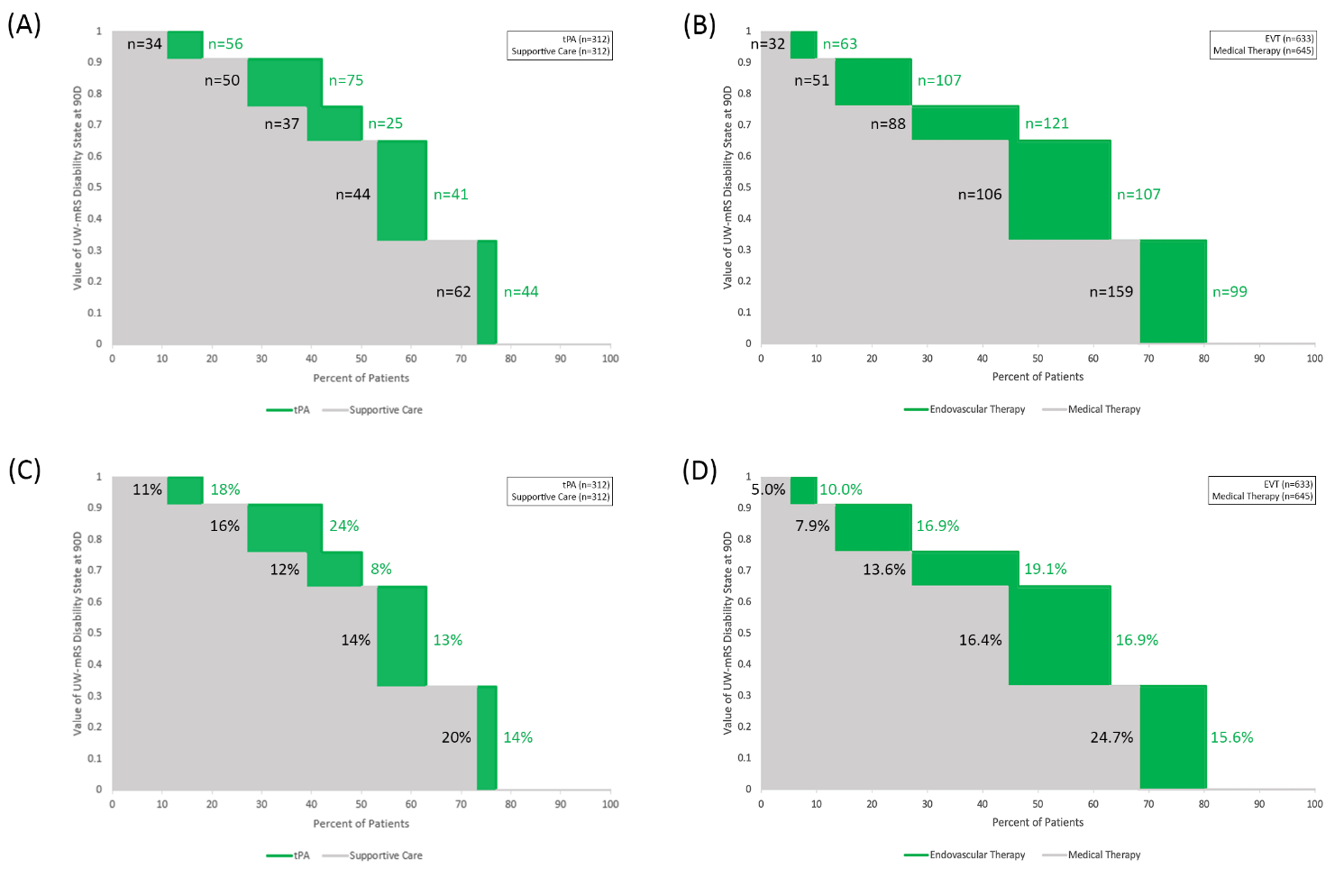
**

**Supplementary Figure 4.** Utility Staircase graphs illustrating the addition of further elements that can optionally be added to Utility Staircase Displays. Top row (A, B) adding the actual n values in each treatment arm as embedded data labels. Bottom row (C, D) adding the percent values in each treatment arm at each mRS level as embedded data labels. Here these elements have been added to the displays of Main Figure 3A and 3G.
